# Adult acute precursor B-cell lymphoblastic leukemia presenting as hypercalcemia and osteolytic bone lesions

**DOI:** 10.1186/s40164-017-0071-8

**Published:** 2017-04-11

**Authors:** Nikki Charlotta Paul Granacher, Zwi N. Berneman, Wilfried Schroyens, Ann L. R. Van de Velde, Anke Verlinden, Alain P. A. Gadisseur

**Affiliations:** Division of Hematology, Antwerp University Hospital, Wilrijkstraat 10, 2650 Edegem, Belgium

**Keywords:** Adult precursor B-cell lymphoblastic leukemia, Case report, Hypercalcemia, Osteolysis, Philadelphia chromosome

## Abstract

**Background:**

Osteolytic bone lesions and hypercalcemia without peripheral blasts B-cell acute lymphoblastic leukemia (B-ALL) is reported in children but rarely seen in adults.

**Case presentation:**

We describe the case of a 34-year old man presenting with hypercalcemia and symptomatic osteolytic bone lesions of vertebrae and ribs who was initially suspected as having a solid malignancy. Diagnostic work-up including peripheral blood examination, radiographic and nuclear studies could, however, not detect a primary tumor. Because of a mild thrombocytopenia and the lack of a primary focus, a bone marrow biopsy was performed leading to the diagnosis of Philadelphia chromosome positive precursor B-ALL. After correction of the hypercalcemia with intravenous fluid administration, corticoids and bisphosphonates, the patient was treated according to the HOVON 100 protocol achieving complete molecular remission after the first cycle of induction chemotherapy.

**Conclusion:**

Hypercalcemia and osteolytic bone lesions are rare complications of adult B-ALL and can occur in the absence of peripheral blastosis. With this case report we would like to emphasize the importance of clinical awareness. Immediate treatment of hypercalcemia and initiation of antileukemic treatment is mandatory as a delay of diagnosis might pose a real and possible life-threatening risk in these patients.

## Background

Hypercalcemia is a common feature of various solid and hematologic malignancies such as multiple myeloma and lymphoma. Underlying mechanisms include either osteolytic bone destruction or tumoral secretion of parathyroid related protein (PTHrP), calcitriol [1,25(OH)vitamine D] and different pro-inflammatory cytokines [tumor necrosis factor alpha (TNF**-**α), interleukins (IL)]. In acute lymphoblastic leukemia (ALL) hypercalcemia and osteolysis are mainly described in children with B-ALL [1–3]. Information regarding adult B-ALL-associated hypercalcemia and osteolytic bone lesions is limited to only a few case reports [4–8].

## Case presentation

**Fig. 1:**
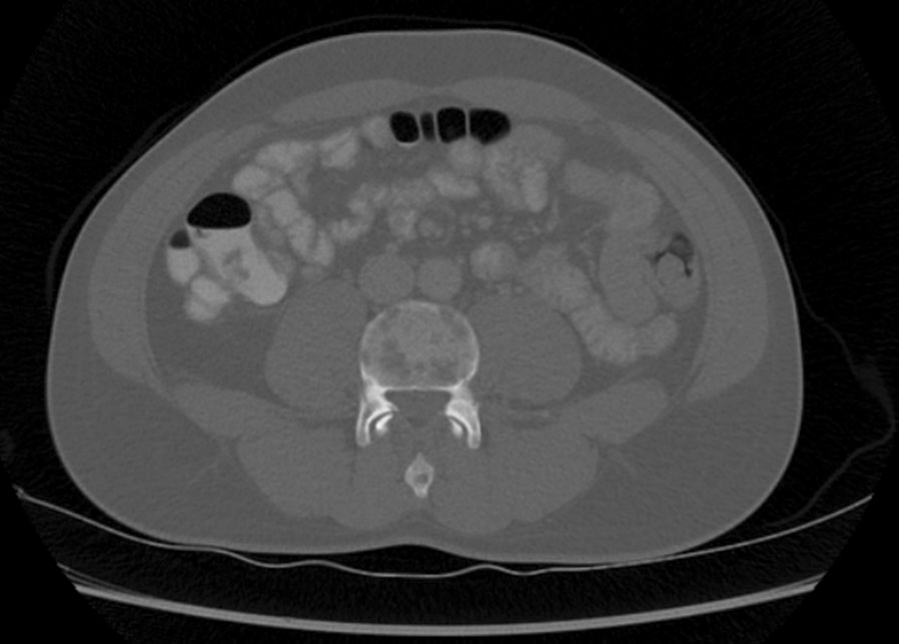
CT scan showing multiple osteolytic lesions of lumbar vertebrae and pelvis

**Fig. 2:**
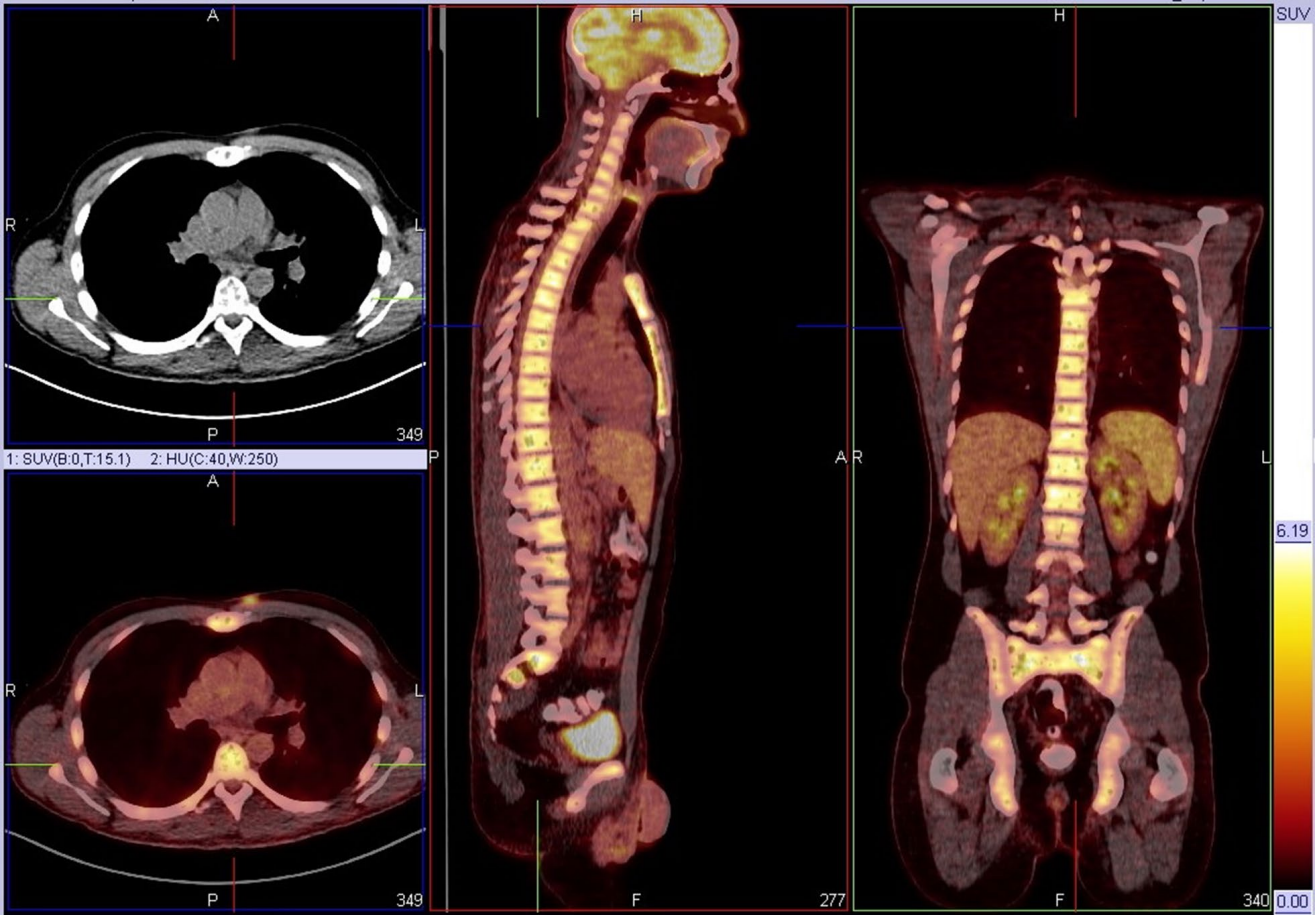
FDG-PET scan revealing diffuse medullar FDG-uptake

A 34-year old man consulted the emergency department because of severe lower back pain. His complaints started 3 weeks earlier, emerging after a day of strenuous physical activity. Despite adequate rest and analgesics the pain gradually increased. At time of presentation in the emergency department there were no other physical complaints, and the patient was on no other medication. Patient’s medical and family history were irrelevant. On clinical examination we only noted diffuse tenderness of the lumbar spine. Laboratory examination revealed a mild thrombocytopenia 79 × 10^9^/l (range 140–440 × 10^9^/l), elevated d-dimers 11.6 ug/ml (normal <0.48 ug/ml), high C-reactive protein 164 mg/l (normal <2.9 mg/l), lactate dehydrogenase 337 U/l (range 84–246 U/l) and marked hypercalcemia 3.2 mmol/l (range 2.1–2.5 mmol/l). Values for hemoglobin, leucocytes, kidney function and liver enzymes were normal. Leucocyte differentiation, however, showed a high concentration of myeloid progenitor cells (11% metamyelocytes; 2.3% myelocytes and 0.5% promyelocytes) in the absence of peripheral blasts. Initial radiographic examination (chest X-ray and lumbar CT scan) showed multiple osteolytic bone lesions of the ribs and lumbar spine (Fig. 1). FDG-PET scan confirmed these bone alterations and displayed diffuse FDG uptake in bone marrow suggestive for a hematologic malignancy (Fig. 2). On bone marrow aspirate and biopsy >50% infiltration with lymphoid blasts was seen with flowcytometric markers compatible with precursor B-ALL (strongly positive for CD10, CD19, CD34, weakly positive for CD33, TdT and CD79a). BCR-ABL p210 fusion proteins were detected (362.7/1000 ABL copies), which was confirmed by the presence of the Philadelphia chromosome t(9,22)(q34,q11,2) on karyotypic examination.

The patient was diagnosed with a Philadelphia chromosome positive precursor B-ALL and was immediately treated with high dose corticosteroids and bisphosphonates (zoledronic acid 4 mg) to reduce hypercalcemia. We initiated treatment with a first generation tyrosine kinase inhibitor imatinib (Glivec 600 mg orally) and a corticosteroid prephase treatment (prednisolone 60 mg/m^2^ for 7 days) within the HOVON 100 collaborative study (hemato-oncologie voor volwassenen Nederland). He was subsequently treated with remission-induction chemotherapy consisting of prednisolone (40 mg/m^2^), daunorubicin (40 mg/m^2^ on day 15 and 22), vincristine (2 mg on day 8, 15, 22 and 29) and PEG-l-asparaginase (1000/m^2^ on day 8 and 21). There were no signs of cerebral invasion on cerebrospinal fluid examination and the patient received on protocol two cycles of prophylactic intrathecal chemotherapy with methotrexate (15 mg) and dexamethasone (4 mg) during remission-induction therapy. He achieved complete molecular remission after the first induction chemotherapy. Because of the Philadelphia chromosome positivity we aimed for an allogeneic stem cell transplantation. However, due to the absence of a suitable HLA-matched donor (sibling or unrelated) and given several therapy related complications in the further course of his treatment (resulting in a moderate general condition of the patient), he eventually proceeded to maintenance chemotherapy.

## Conclusion

Osteolytic bone lesions and hypercalcemia are a rare presentation of adult B-ALL with only a few cases reported worldwide (Table 1) [4–8]. Searching literature we found five reports in total (concerning 6 patients) with only a single case of a solitary osteolytic lesion (mandibular) [4]. The majority of reports describe diffuse osteolytic lesions in newly diagnosed B-ALL as was seen in our patient [5–8]. Two cases present the occurrence of bone lesions in relapsed disease [4, 6].

**Table 1:** published case reports concerning B-ALL, osteolysis and hypercalcaemia

Author	Patient	Diagnosis	Osteolytic lesions	Mechanism
Chung [4]	Male 35 years	B-ALL Burkitt type	Left mandibula	PTHrP elevated
Seymour [7]	Female 65 years	B-ALL relapse	Multiple lesions	PTHrP nl
	Female 44 years	B-ALL	Mulitple lesions	PTHrP nl
Kaiafa [6]	Male 24 years	Pre-B ALL relapse with lineage switch	Multiple lesions	PTHrP nl PTH low 1,25(OH)D nl
Verma [8]	Female 27 years	Pre-B ALL	Multiple skull lesions	N/A
Fukasawa [5]	Female 53 years	Pre-B ALL	Multiple lesions	PTH low PTHrP nl 1,25(OH)D low TNF**-**α elevated IL-6 elevated Solube IL-2 elevated

*B*-*ALL* acute B-cell lymphoblastic leukemia, *PTHrP* parathyroid hormone related protein, *PTH* parathyroid related hormone, *TNF* **-** *α* tumor necrosis factor alpha, *IL6* interleukin 6, *nl* normal, *N/A* not available

Most information regarding B-ALL associated hypercalcemia and osteolysis comes from reports in pediatric patients in whom radiographic bone alterations can be found in 21–55% at time of diagnosis. However, severe osteolysis in children with B-ALL is also rare and the described alterations mostly concern osteopenia or periosteal reactions [1, 3]. Concomitant hypercalcemia in children is even less common with a reported incidence varying between 0.6 and 4.8%. This hypercalcemic subset of patients seems characterized by relatively older age (10–20 years whereas typical age lies between 4 and 14 years), ‘aleukemic’ presentation (normal white blood cell count without peripheral blastosis) and the absence of organomegaly and lymphadenopathy. On bone marrow examination less common immunophenotypes of leukemic blasts (CD10 positive and CD19 negative) have been observed [2, 9]. In a pediatric retrospective study performed by Inukai and colleagues 22 cases of childhood B-ALL related hypercalcemia were analyzed (n = 18 newly diagnosed and n = 4 relapsed disease). The majority of patients only had mild deviations in hemoglobin, leukocyte and platelet count and peripheral blasts were absent in 8 patients. Inukai and colleagues found an association between the translocation t(17,19) and the occurrence of hypercalcemia. In general, t(17,19) resulting in the E2A-HLF fusion transcription factor exhibits only 1% of childhood B-ALL whereas Inukai and collegues found this translocation in 5 of the 17 analyzed cases [2]. Additional investigations are needed to fully outline and understand this association.

The underlying mechanism of hypercalcemia and osteolysis in B-ALL is not completely understood. In general, malignant hypercalcemia is caused by either local bone destruction by tumoral medullar/bone invasion or by increased osteoclast activity through the production of humoral tumor-derived factors (e.g. PTHrP, IL1, IL6, IL11 and TNF**-**α and beta). Concerning B-ALL, lymphoblasts have been shown to produce PTHrP [2, 10, 11]. PTHrP stimulates bone resorption, renal calcium resorption and renal phosphate excretion via binding to the PTH/PTHrP-receptor. Higher serum levels have been detected in the majority of pediatric leukemic patients [2]. PTHrP levels have also been found to be normal, suggesting the importance of other humoral factors in this process [5–7]. For example: Fukasawa et al. demonstrated elevated serum concentrations of TNF**-**α, IL-6 and soluble IL-2 but normal concentration of PTHrP. Induction chemotherapy resulted in normocalcemia and a decrease in serum levels of the above mentioned cytokines [5].

In conclusion we can state that osteolytic bone lesions and hypercalcemia as sole presentation of B-ALL in adults is extremely rare with only few cases reported worldwide. As underlying mechanisms the local lymphoblast production of several humoral tumor-derived factors (PTHrP, IL-1, IL-6, IL-11, TNF**-**α and beta) is postulated. Since we did not perform PTHrP or IL testing, the pathophysiology in our patient cannot be established. Immediate recognition and treatment of hypercalcemia and the underlying B-ALL is vital since a delay of diagnosis poses a possible life-threatening risk.

## Abbreviations

B-ALL: acute B-cell lymphoblastic leukemia
T-ALL: acute T-cell lymphoblastic leukemia
ALL: acute lymphoblastic leukemia
PTHrP: parathyroid hormone related protein
PTH: parathyroid related hormone
TNF**-**α: tumor necrosis factor alpha
IL: interleukins
IL6: interleukin 6
nl: normal
N/A: not available
